# Supplementary material for: Mitochondrial DNA is a target of HBV integration
Source: Commun Biol. 2023 Jul 3;6:684. doi: 10.1038/s42003-023-05017-4 (PMC10318008; doi:10.1038/s42003-023-05017-4)
Supplement: Supplementary file 2 — Supplementary Information [file 42003_2023_5017_MOESM2_ESM.pdf]

Supplementary Information

Mitochondrial DNA is a target of HBV integration

Domenico Giosa, Daniele Lombardo, Cristina Musolino, Valeria Chines, Giuseppina Raffa, Francesca Casuscelli di Tocco, Deborah D'Aliberti, Giuseppe Caminiti, Carlo Saitta, Angela Alibrandi, Riccardo Aiese Cigliano, Orazio Romeo, Giuseppe Navarra, Giovanni Raimondo, Teresa Pollicino.

INDEX

Supplementary Table 1

Supplementary Table 2

Supplementary Table 3

Supplementary Figure 1

Supplementary Figure 2

Supplementary Figure 3

Supplementary Figure 4

Supplementary Figure 5

Supplementary Figure 6

Supplementary Figure 7

Supplementary Figure 8

Supplementary Figure 9

Supplementary Figure 10

Supplementary Figure 11

Supplementary Figure 12

Supplementary Figure 13

Supplementary References

The following documents are provided as separate files:

Supplementary Data 1

Supplementary Data 2

Supplementary Data 3

Supplementary Data 4

Supplementary Data 5

Supplementary Table 1. **Microhomology (MH) sequences (3bp-15bp in length) between cellular DNA and integrated HBV DNA at insertion sites (IS) in non-tumour and tumour tissue samples.**

Microhomology (MH) sequence length	MH sequences at IS in non-tumour tissue samples			MH sequences at IS in tumour tissue samples		
	Counts	Ratio (%) of total IS (426)	P-value	Counts	Ratio (%) of total IS (2,913)	P-value
**≥ 3bp***	322	75.6	<0.0001	2,368	81.3	<0.0001
**≥ 5bp**	295	69.2	<0.0001	2,167	74.4	<0.0001
**≥ 7bp**	262	61.5	<0.0001	1,929	66.2	<0.0001
**≥ 9bp**	207	48.6	<0.0001	1,331	45.7	<0.0001
**≥ 11bp**	136	31.9	<0.0001	833	28.6	<0.0001
**≥ 13bp**	105	24.6	=0.024	640	22.0	<0.0001
**≥ 15bp**	70	16.4	=0.46	489	16.8	=0.0001

*bp: base pair

Supplementary Table 2. Sequences of oligonucleotide primers used in the study

Oligonucleotide primers reported in a, b, and c panels were used for integration library construction

a

Name	Sequence primer (5’->3’)
pLinkerTop	GCAGCGGATAACAATTTCACACAGGACGTACTGTGGCGCGCCT
pLinkerBottom	Ph – GGCGCGCCACAGTACTTGACTGAGCTTTA – ddC
pLinker	GCAGCGGATAACAATTTCACACAGGAC

b

Name	Polarity	Sequence primer (5’->3’)	Position°
B-HBXF	Forward	B - TGCCAAGTGTTTGCTGACGCAAC	1176-1198
B-HB3XR	Reverse	B - TGAAGGAAAGAAGTCAGAAGG	1980-1960
B-HBXR	Reverse	B - CTAGGAGTTCCGCAGTATGGATC	1287-1265
B-HBXAR	Reverse	B - TGAGTGCAGTATGGTGAGG	2068-2050
B-HBXBF	Forward	B - CGGGGCGCACCTCTCTTTACG	1522-1542
B-HBXCF	Forward	B - CCATACTGCGGAACTCCTAGC	1268-1288
B-HBCore F	Forward	B - CCTAGAAGAAGAACTCCCTC	2368-2387
B-HBCore R	Reverse	B - TTATGAGTCCAAGGAATACTAAC	2472-2450
B-HBSF	Forward	B - TTGGGGTGGAGCCCTCAGGCT	3039-3059
B-HBSR	Reverse	B - AGGGGTCCTAGGAATCCTGATG	191-170
B-HBS1F	Forward	B - CCTGCTGGTGGCTCCAGTTCA	58-78
B-HBS2F	Forward	B - TATGCCTCATCTTCTTGTTGGTTC	422-445
B-HBS3R	Reverse	B - CTTGGCCCCCAATACCACATC	767-748
B-HBPreSF	Forward	B - GGTCACCATATTCTTGGGAA	2817-2836
B-HBPreSR	Reverse	B - TTGAAGTCCCAATCTGGATT	2953-2934
B-HB3XF	Forward	B - GCTACTGTGGAGTTACTCTCG	1933-1953
B-HBXDR	Reverse	B - AGAAGGCACAGACGGGGAG	1565-1547
B-HBP24R	Reverse	B - ACCCAAGGCACAGCTTGGAGG	1891-1871
B-HBP26F	Forward	B - CACCTCTGCCTAATCATCTCWT	1828-1849
B-HBS4R	Reverse	B - ACAAACGGGCAACATACCTTGA	456 – 477
B-HBS5F	Forward	B - GCTCAGTTTACTAGTGCCATT	672 - 692

W = A or T according to IUPAC nucleotide code. bp, base pair; B = Biotin. ° Nucleotide positions of the primers are numbered from the unique *Eco*RI site and the nomenclature is according to to the GenBank reference sequence NC_003977.2

c

**Name**	**Polarity**	**Sequence primer (5’->3’)**
pLinker F	Forward	*Adapter1 - GCAGCGGATAACAATTTCACACAGGAC
PLinker R	Reverse	*Adapter2 – GCAGCGGATAACAATTTCACACAGGAC

**Name**	**Polarity**	**Sequence primer (5’->3’)**	**Position°**
MiSeq HBXF	Forward	*Adapter1 - TGCCGATCCATACTGCGGAAC	1261-1281
MiSeq HB3XR	Reverse	*Adapter2 - CGAGAGTAACTCCACAGTAGC	1953-1933
MiSeq HBXR	Reverse	*Adapter2 - GGTTGCGTCAGCAAACACTTGG	1199-1178
MiSeq HBXAR	Reverse	*Adapter2 - TGCTCAGGAGACTCTAAGGC	2042-2023
MiSeq HBXBF	Forward	*Adapter1 - CTCCCCGTCTGTGCCTTCT	1547-1565
MiSeq HBXCF	Forward	*Adapter1 - CCATGGCTGCTAGGCTGTGCTGCCAAC	1374-1400
MiSeq HBCoreF	Forward	*Adapter1 - GTCGCAGAAGATCTCAATC	2417-2435
MiSeq HBCoreR	Reverse	*Adapter2 - AGATTGAGATCTTCTGCGACG	2436-2416
MiSeq HBSF	Forward	*Adapter1 - CTGCTGGTGGCTCCAGTTCA	59-78
MiSeq HBSR	Reverse	*Adapter2 - TGAACTGGAGCCAGCAGCAGG	78-58
MiSeq HBS1F	Forward	*Adapter1 - CATGGAGAACATCACATCAGGA	156-177
MiSeq HBS2F	Forward	*Adapter1 - TCAAGGTATGTTGCCCGTTTGT	456-477
MiSeq HBS3R	Reverse	*Adapter2 - AATGGCACTAGTAAACTGAGC	692-672
MiSeq HBPreSF	Forward	*Adapter1 - CTCTGGGATTCTTTCCCGA	2878-2896
MiSeq HBPreSR	Reverse	*Adapter2 - GTCGGGAAAGAATCCCAGAGG	2897-2877
MiSeq HB3XF	Forward	*Adapter1 - CCTTCTGACTTCTTTCCTTCA	1960-1980
MiSeq HBXDR	Reverse	*Adapter2 - CGTAAAGAGAGGTGCGCCCCG	1542-1522
MiSeq HBP24R	Reverse	*Adapter2 - AWGAGATGATTAGGCAGAGGTG	1849-1828
MiSeq HBP26F	Forward	*Adapter1 - CCTCCAAGCTGTGCCTTGGGT	1871-1891
MiSeq HBS4R	Reverse	*Adapter2 - GGAACCAACAAGAAGATGAGGCATA	422 – 445
MiSeq HBS5F	Forward	*Adapter1 - CGATGTGGTATTGGGGGCCAAG	747 – 767

W = A or T according to IUPAC nucleotide code. bp, base pair. *Adapter 1 and 2 are from Illumina technology. °Nucleotide positions of the primers are numbered from the unique EcoRI site and the nomenclature is according to the GenBank reference sequence NC_003977.2

Primers for validation of HBV-host fusions

Fusion partners	Nested-PCR	Forward Primer (5'->3')	Reverse Primer (5'->3')	Product Size (bp)
**ND5** – HBx	1st round	**CTCATGTTCATACACCTATCCC**	GTTGGCGAGAAAGTGAAAGCC	616
	2nd round	**GTTCATACACCTATCCCCCAT**	GCGAGAAAGTGAAAGCCTGCT	608
	1st round	TGGTTATCCTGCKTTRATGCC	**CTCGGGCGTATCATCAACTGA**	435
	2nd round	CCTGCKTTRATGCCWTTGT	**ATAATTCCTACGCCCTCTCAG**	363
HBs – **Dloop**	1st round	CCTCTGGGATTCTTTCCCGA	**TAGGGGAACGTGTGGGCT**	455
	2nd round	TGGGATTCTTTCCCGACCAC	**GGAACGTGTGGGCTATTTA**	449
HBs – **Dloop**	1st round	CCTCTGGGATTCTTTCCCGA	**TAGGGGAACGTGTGGGCT**	350
	2nd round	TGGGATTCTTTCCCGACCAC	**GGAACGTGTGGGCTATTTA**	345
HBp – **RNR2**	1st round	GCAGGACCCCTAGAAGAAGAACT	**ATGAGCATGCCTGTGTTGGG**	408
	2nd round	CCCTAGAAGAAGAACTCCCTC	**CCTGTGTTGGGTTGACAGTGA**	392
**Cytb –** HBs	1st round	**TATCCGCCATCCCATACATT**	AGTTTGTAGTATGCCCTGAGC	338
	2nd round	**CATTGGGACAGACCTAGTTC**	GTCTGGCCAAGGGTCCTTGTTG	223
**RNR2** – HBx	1st round	**CTCTGAGCTAAACCTAGC**	GTGGGGGTTGCGTCAGCAAACA	406
	2nd round	**GAGCTAAACCTAGCCCCAAAC**	TTGCGTCAGCAAACACTTGGC	397
**RNR2** – HBx	1st round	**CTCTGAGCTAAACCTAGC**	GTGGGGGTTGCGTCAGCAAACA	239
	2nd round	**GAGCTAAACCTAGCCCCAAAC**	TTGCGTCAGCAAACACTTGGC	230
HBc – **COX1**	1st round	CCAAGCTGTGCCTTGGGT	**GAGAGATAGGAGAAGTAG**	382
	2nd round	TGTGCCTTGGGTGGCTTTGG	**AGATAGGAGAAGTAGGAC**	377
HBc – **COX1**	1st round	CCAAGCTGTGCCTTGGGT	**GAGAGATAGGAGAAGTAG**	492
	2nd round	TGTGCCTTGGGTGGCTTTGG	**AGATAGGAGAAGTAGGAC**	488
**BEND3** – HBx	1st round	**ACCTCGGCTCTCTGCAACT**	GTCTGTGCCTTCTCATCTGCC	199
	2nd round	**CTCTCTGCAACTCTGCATCC**	TTCTCATCTGCCGGACCGTGT	183
**EXOC4** – HBx	1st round	**TGAGATGGAGCCTCACTCCAT**	GTCTGTGCCTTCTCATCTGCC	169
	2nd round	**CTCACTCCATCGCCCAGGTT**	TTCTCATCTGCCGGACCGTGT	149
HBx – **MTHFS**	1st round	CTGCCGATCCATACTGCGGAA	**TCACTCCTGAATTCTTTCCCG**	249
	2nd round	CCATACTGCGGAACTCCTAGC	**TGAATTCTTTCCCGGCAAAGC**	234
**PCNX2** – HBc	1st round	**CCAGGCATATTTAGAGGC**	ACCCAAGGCACAGCTTGG AGG	154
	2nd round	**CATATTTAGAGGCTTGGTGAGG**	ACAGCTTGGAGGCTTGAAC	145
HBx – **CCPG1**	1st round	GACCTTGAGGCATACTTC	**GCATTCTTTCTCCACTGTGTG**	677
	2nd round	GAGGCATACTTCAAAGACTG	**CTCCACTGTGTGAAAGCTTC**	663
HBx – **-180572 RPL29P32**	1st round	GACCTTGAGGCATACTTC	**CTCAGTTTTAGGCCCTCCCTT**	177
	2nd round	GAGGCATACTTCAAAGACTG	**TTAGGCCCTCCCTTTAGGCT**	164
HBx – **NALCN-AS1**	1st round	CTGTAATGTCAACGACCGAC	**CTTCTGGGATAAAATGAC**	176
	2nd round	ATGTCAACGACCGACCTT	**ACATTTCAGTAAAGACCC**	155
**PIKFYVE** – HBs	1st round	**TGCCCGTTTGTGTATGGC**	GTCCTAGGAATCCTGATGTGA	157
	2nd round	**CCGTTTGTGTATGGCTTGTG**	TCCTGATGTGATGTTCTCCATG	144

**DOCK3** – HBs	1st round	**GATATGCCAAACATGGGGTG**	TCCTGATGTGATGTTCTCCATG	500
	2nd round	**GGTGGATTATTCATGCCTCCT**	TGTGATGTTCTCCATGTTCAG	478
**ZNF644-FAM101A** – HBs	1st round	**CGTTTGTCAGTTCTGATTTG**	TCCTGATGTGATGTTCTCCATG	217
	2nd round	**GTGCTTAAGAGCAAAGGTTC**	TGTGATGTTCTCCATGTTCAG	181
**LIMS3** – HBc	1st round	**CTGGCGCAGAGACAAGAATC**	AGATGATTAGGCAGAGGTGAAAAA	191
	2nd round	**ATCAAGGGGTGCTGAATCTAG**	AAAAAGTTGCATGGTGCTGG	156
**-91907 LOC105377508** – HBs	1st round	**TTTGGAAACTGGGCTATTG**	CCTGCTGGTGGCTCCAGTT	107
	2nd round	**AAACTGGGCTATTGATATC**	TGGTGGCTCCAGTTCAGG	97
**hTERT** – HBx	1st round	**CCCACTGGGACCTGAAGC**	CTGCCGATCCATACTGCGGA	192
	2nd round	**CTGGGACCTGAAGCCTGCAGC**	ATCCATACTGCGGAACTCC	182
HBs – **CCDC57**	1st round	TATGCCTCATCTTCTTGTTGG	**CCTCACCTGCTAAAGTCTCCT**	298
	2nd round	GGTTCTTCTGGACTATCAAGGT	**CCTCACCTGCTAAAGTCTCCT**	279

Specific oligonucleotide sequences used for Real-time PCR of HBV integration in mitochondrial RNR2 gene and D-Loop

**Name**	**Polarity**	**Sequence primer (5'->3')**	**mtDNA Position***	**HBV Position°**
RNR2_intF_1	Forward	GCAGGTCCCCTAGAAGAAGAACT		2360-2382
**RNR2_intR_1**	Reverse	**ATGAGCATGCCTGTGTTGGG**	**2445-2426**	
RNR2_intF_2	Forward	CCCTAGAAGAAGAACTCCCTC		2367-2387
**RNR2_intR_2**	Reverse	**CCTGTGTTGGGTTGACAGTGA**	**2436-2416**	
D-Loop_intF_1	Forward	CCTCTGGGATTCTTTCCCGA		2877-2896
**D-Loop_intR_1**	Reverse	**TAAGGGGAACGTGTGGGCT**	**16552-16534**	
D-Loop_intF_2	Forward	TGGGATTCTTTCCCGACCAC		2881-2900
**D-Loop_intR_2**	Reverse	**GGAACGTGTGGGCTATTTA**	**16547-16529**	
**mtDNA_wtF_1**	Forward	**CGTGAAATCAATATCCCGCA**	**16411-16430**	
**mtDNA_wtR_1**	Reverse	**AAATTTGAAATCTGGTTAGG**	**400-381**	
**mtDNA_wtF_2**	Forward	**CAATATCCCGCACAAGAGTGC**	**16419-16439**	
**mtDNA_wtR_2**	Reverse	**TAGGCTGGTGTTAGGGTTCT**	**384-365**	

mtDNA, mitochondrial DNA, * Nucleotide positions of the primers are in accordance to the GenBank reference sequence J01415.2. °Nucleotide positions of the primers are numbered from the unique EcoRI site and the nomenclature is according to the GenBank reference sequence NC_003977.2.

Specific oligonucleotide sequences used for amplification of full-lenght HBV DNA and cccDNA

Name	Polarity	Sequence primer (5'->3')	Position°
FLHBVF	Forward	CCAGCACCATGCAACTTTTT	1808-1827
FLHBVR	Reverse	AAAAAGTTGCATGGTGCTGG	1827-1808
cccDNAF	Forward	CGGGGCGCACCTCTCTTTACG	1522-1542
cccDNAR	Reverse	GCCCCAAAGCCACCCAAG	1885-1902

°Nucleotide positions of the primers are numbered from the unique EcoRI site and the nomenclature is according to the GenBank reference sequence NC_003977.2.

Specific oligonucleotide primer and probe sequences used for real time PCR quantification of HBV pgRNA, cccDNA, and open circular DNA

Name	Polarity	Sequence primer (5'->3')	Position°
pgRNAF	Forward	CAAGCCTCCAAGCTGTGCCTTG	1867-1888
pgRNAR	Reverse	GGAAAGAAGTCAGAAGGCAAAAACG	1976-1952
pgRNAprobe	Forward	6FAM-ATAAAGAATTTGGAGCTACTGTGGAGTTAC-BHQ1	1919-1948
cccDNA2F	Forward	CTCCCCGTCTGTGCCTTCT	1547-1565
cccDNA2R	Reverse	GCCCCAAAGCCACCCAAG	1902-1885
cccDNAprobe	Reverse	6FAM-CATGCGACGTGCAGAGGTGAAGCGAA-TMR	1612-1587
ocHBVF	Forward	CTCGTGGTGGACTTCTCTC	255-273
ocHBVR	Reverse	CAGCAGGATGAAGAGGAA	420-403
ocHBVprobe	Reverse	6FAM-AACCAGGACAAATTGGAGGACAGGA-TMR	370-346

°Nucleotide positions of the primers are numbered from the unique EcoRI site and the nomenclature is according to the GenBank reference sequence NC_003977.2.

Specific oligonucleotide sequences used for PCR amplification of HBV transcripts in isolated mitochondria

Name	Polarity	Sequence primer (5'->3')	Position°
HBPreSF	Forward	GGTCACCATATTCTTGGGAA	2817-2836
HBPreSREV	Reverse	GACATACTTTCCAATCAATAG	993-973
HBS4F	Forward	AATCCAGATTGGGACTTCAA	2934-2953
HBS4R	Reverse	ACCTTGATAGTCCAGAAGAACC	462-441
HBX4F	Forward	ACGTCCTTTGTTTACGTCCCG	1416-1436
HBX4R	Reverse	TGCGCAGACCAATTTATGCCTACAGC	1807-1782
HBεF	Forward	CACCTCTGCCTAATCATC	1828-1845
HBεF	Reverse	TGAGTGCAGTATGGTGAGG	2068-2050

°Nucleotide positions of the primers are numbered from the unique EcoRI site and the nomenclature is according to the GenBank reference sequence NC_003977.2.

Specific oligonucleotide primer sequences used for amplification of mitochondrial genome

Name	Polarity	Sequence primer (5'->3')	Position*
COX3_1F	Forward	AAAGCACATACCAAGGCCAC	9397-9416
COX3_1R	Reverse	AATGTTGAGCCGTAGATGCC	9796-9777

* Nucleotide positions of the primers are in accordance to the GenBank reference sequence J01415.2.

Specific oligonucleotide sequences used for amplification of human control gene

Name	Polarity	Sequence primer (5'->3')	Position[&]
GAPDH_1F	Forward	TGCACCACCAACTGCTTAGC	530-549
GAPDH_1R	Reverse	GGCATGGACTGTGGTCATGAG	616-596

[&]Nucleotide positions of the primers are in accordance to the GenBank reference human transcript sequence NM_002046.7

Specific oligonucleotide primer sequences used for construction of HBV vector plasmids

Name	Polarity	Sequence primer (5'->3')	Position°
PreS1_vector_F	Forward	AAGGTACCAGGCATGGGGCAGAAT	2850-2861
PreS1_vector_R	Reverse	GAGCGGCCGCCACCGTTAAATGTATACCCA	837-823
SLα_vector_F	Forward	AAGGTACCAGGCTGGCTCAGTTTAC	670-681
SLα _vector_R	Reverse	GAGCGGCCGCCACCGGGACAACAGAGTTAT	1350-1336
HBX_vector_F	Forward	AAGGTACCAGGCATGGCTGCTAGGCTG	1376-1390
HBX_vector_R	Reverse	GAGCGGCCGCCACCGTTAGGCAGAGGTGAA	1840-1826
HBε_vector_F	Forward	AAGGTACCAGGCGGCTGTAGGCAT	1781-1792
HBε_vector_R	Reverse	GAGCGGCCGCCACCGTGAGTGCAGTATGGT	2068-2054
COX3_vector_F	Forward	TTGGTACCGGGCAAAGCACATACCAAGGCCAC	9397-9416
COX3_vector_R	Reverse	GAGCGGCCGCCACCGAATGTTGAGCCGTAGATGCC	9796-9777

Underlined sequence: Kpn I restriction site. Dashed sequence: Not I restriction site. °Nucleotide positions of the primers are numbered from the unique EcoRI site and the nomenclature is according to the GenBank reference sequence NC_003977.2.

Specific oligonucleotide sequences used for amplification of NTCP

Name	Polarity	Sequence primer (5'->3')	Position#
NTCP_1F	Forward	CTGGCACTGAGCGTCATCCT	159-178
NTCP_1R	Reverse	ATGGCCAGCCCTTTAGGCTT	274-255

Nucleotide positions of the primers are in accordance to the GenBank reference human transcript sequence NM_003049.4

Supplementary Table 3. HBV integrated sequences into the genome of PLC/PRF/5 cell line[#]

Human Chromosome (Chr)*	Human Junction Nucleotide Position	Gene Name	HBV Fragment Start Position	HBV Fragment End Positiion	HBV Genome
**Chr. 1**	143265963	LOC101929814	2877	3081	PreS/S
**Chr. 2**	101028487	TBC1D8	1374	1403	X
**Chr. 3**	131451702	LOC105374110	1260	1408	ENH1/X promoter/X
**Chr. 4**	145300125	RPS23P4	1374	1413	X
**Chr. 5**	1297478	TERT	1260	1391	ENH1/X promoter/X
**Chr. 8**	35446428	UNC5D	2436	2392	PreC/C
**Chr. 8**	98178750	LOC105375660	2399	2436	PreC/C
**Chr. 8**	117239293	LOC105375716	1374	1403	X
**Chr. 12**	109573983	MVK	711	817	PreS/S
**Chr. 13**	33088561	STARD13	2138	2057	PreC/C
**Chr. 17**	82105784	CCDC57	491	456	PreS/S
**Chr. 20**	25566052	NINL	1374	1404	X

[#]All these HBV integrations were covered by at least 3 reads

*Chr., chromosome (NCBI assembly GRCh38.p14); TBC1D8, TBC1 Domain Family Member 8; TERT, Telomerase Reverse Transcriptase; UNC5D, unc-5 netrin receptor D; MVK, mevalonate kinase; STARD13, STAR Related Lipid Transfer Domain Containing 13; CCDC57, Coiled-Coil Domain Containing 57, HBV, hepatitis B virus.

Supplementary Figure 1

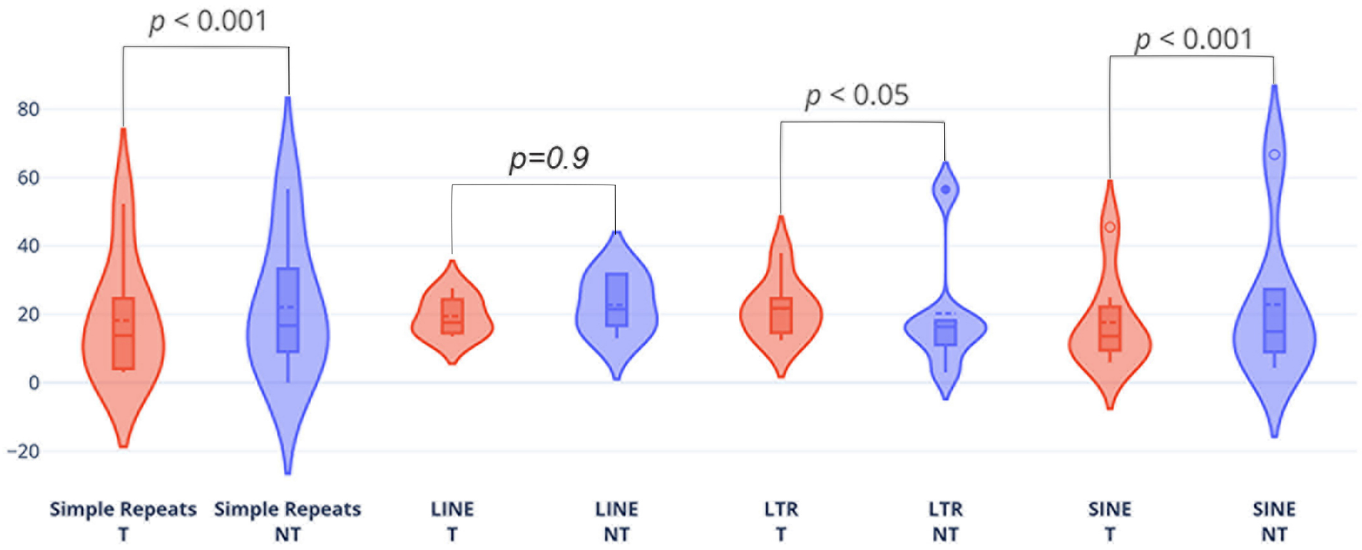

Supplementary Fig. 1. Distribution of HBV integrations sites in different genomic elements. Violin plot of HBV integration sites detected in LINE (long interspersed nuclear elements), LTR (long terminal repeats) SINE (Short Interspersed Nuclear Elements) from tumour (T) and non-tumour (NT) tissues. Boxes in violin plots extend from 25th to 75th percentiles. Solid line denotes the median, dashed line denotes the mean (Fisher's exact test).

Supplementary Figure 2

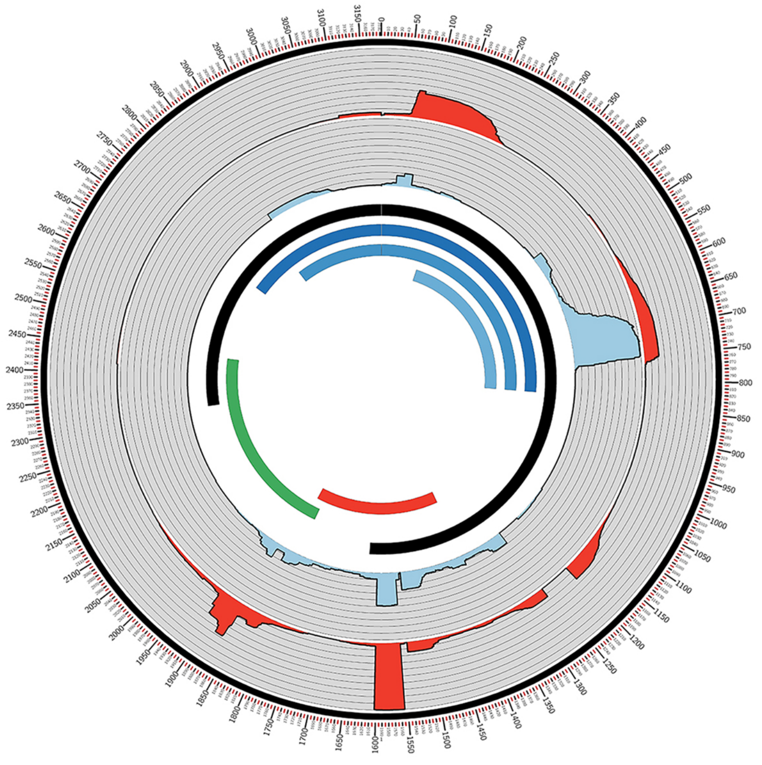

Supplementary Fig. 2. **Distribution of integration breakpoints in the HBV genome.** Distribution of integration breakpoints in the HBV genome in tumour and non-tumour samples represented in circos plot. Each bar denotes the number of HBV integration breakpoints (tumour: red; non-tumour: blue) at a particular site in the HBV genome. Outer DNA numbering is given in bases. HBV open reading frames are represented in black (Polymerase), different gradients of blue (preS/S), green, (Precore/Core), and red (X).

Supplementary Figure 3

a

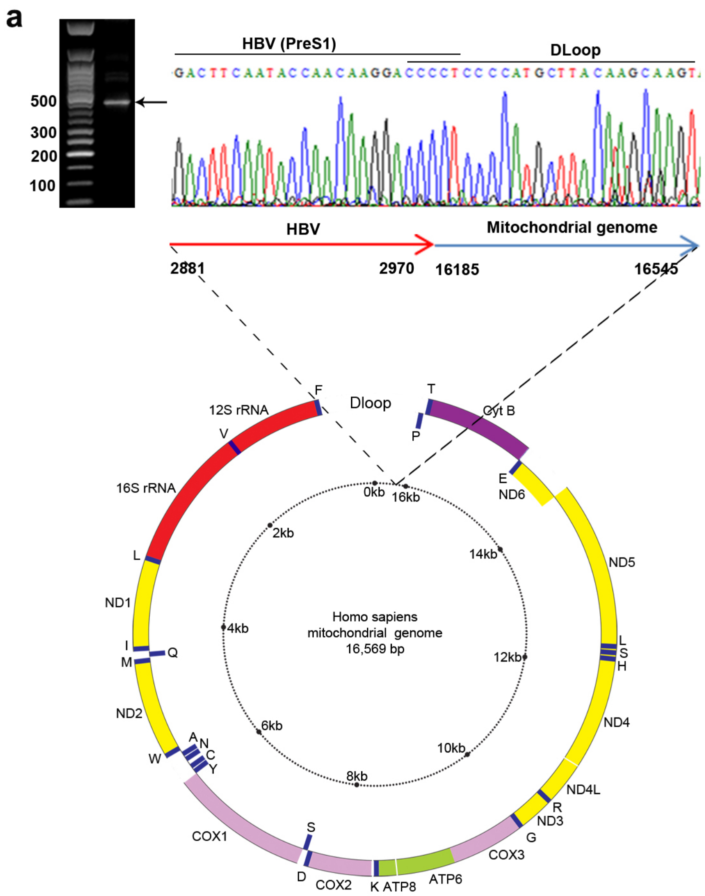


```
  1 TGGGATTCTT TCCCGACCAC CAGTTGGATC CAGCCTTCAG  40
 41 AGCAAACACC GCAAATCCAG ATTGGGACTT CAATACCAAC  80
 81 AAGGACCCCT CCCCATGCTT ACAAGCAAGT ACAGCAATCA 120
121 ACCCTCAACT ATCACACATC AACTGCAACT CCAAAGCCAC 160
161 CCCTCACCCA CTAGGATACC AACAAACCTA CCCACCCTTA 200
201 ACAGTACATA GCACATAAAG CCATTTACCG TACATAGCAC 240
241 ATTACAGTCA AATCCCTTCT CGTCCCCATG GATGACCCCC 280
281 CTCAGATAGG AGTCCCTTGA CCACCATCCT CCGTGAAATC 320
321 AATATCCCGC ACAAGAGTGC TACTCTCCTC GCTCCGGGCC 360
361 CATAACACTT GGGGGTAGCT AAAGTGAACT GTATCCGACA 400
401 TCTGGTTCCT ACTTCAGGGT CATAAAGCCT AAATAGCCCA 440
441 CACGTT 446
```

b

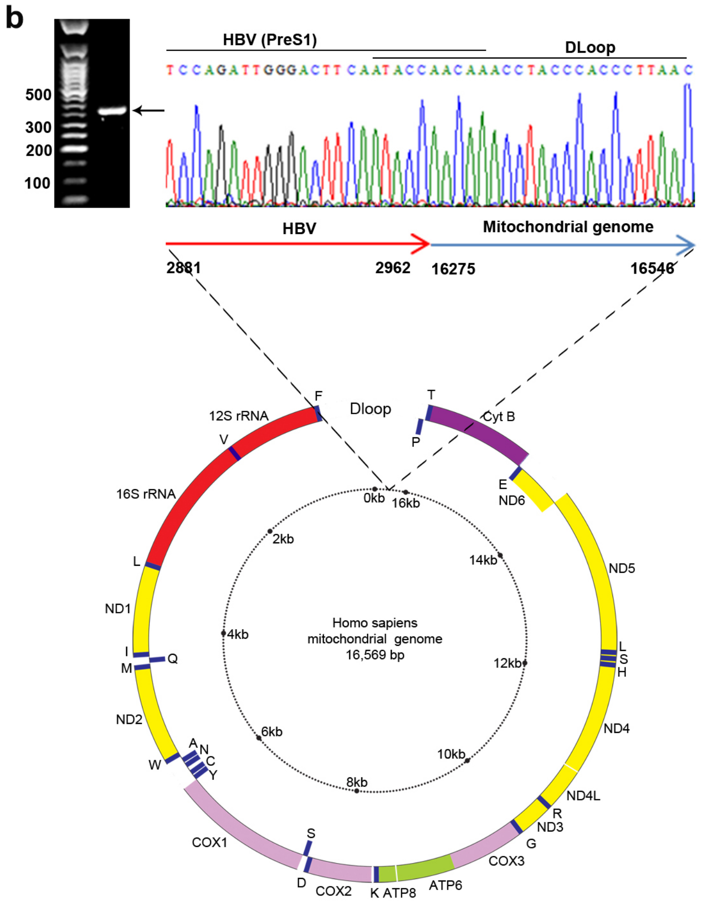


```
  1 TGGGATTCTT TCCCGACCAC CAGTTGGATC CAGCCTTCAG  40
 41 AGCAAACACC GCAAATCCAG ATTGGGACTT CAATACCAAC  80
 81 AAACCTACCC ACCCTTAACA GTACATAGCA CATAAAGCCA 120
121 TTTACCGTAC ATAGCACATT ACAGTCAAAT CCCTTCTCGT 160
161 CCCCATGGAT GACCCCCCTC AGATAGGAGT CCCTTGACCA 200
201 CCATCCTCCG TGAAATCAAT ATCCCGCACA AGAGTGCTAC 240
241 TCTCCTCGCT CCGGGCCCAT AACACTTGGG GGTAGCTAAA 280
281 GTGAACTGTA TCCGACATCT GGTTCCTACT TCAGGGTCAT 320
321 AAAGCCTAAA TAGCCCACAC GTTC 344
```

Supplementary Fig.3. Verification of HBV integration in the *D-loop* region of mitochondrial DNA from patient 0504 by PCR amplification and Sanger sequencing. Panels **a** and **b** show two different HBV integration sites in the mitochondrial *D-loop* genomic region. Electropherograms detail sequences of the HBV *preS-D-Loop* junctions. The coordinates of the inserted HBV nucleotide fragments are given relative to the reference sequence NC_003977.2. Nucleotide position at the mitochondrial (GenBank:J01415.) insertion sites are also indicated. In the HBV-mitochondrial fusion sequence, the HBV nucleotide sequence is shown in red and the mitochondrial sequence in blue. The overlapping microhomology sequences are highlighted in black.

Supplementary Figure 4

a

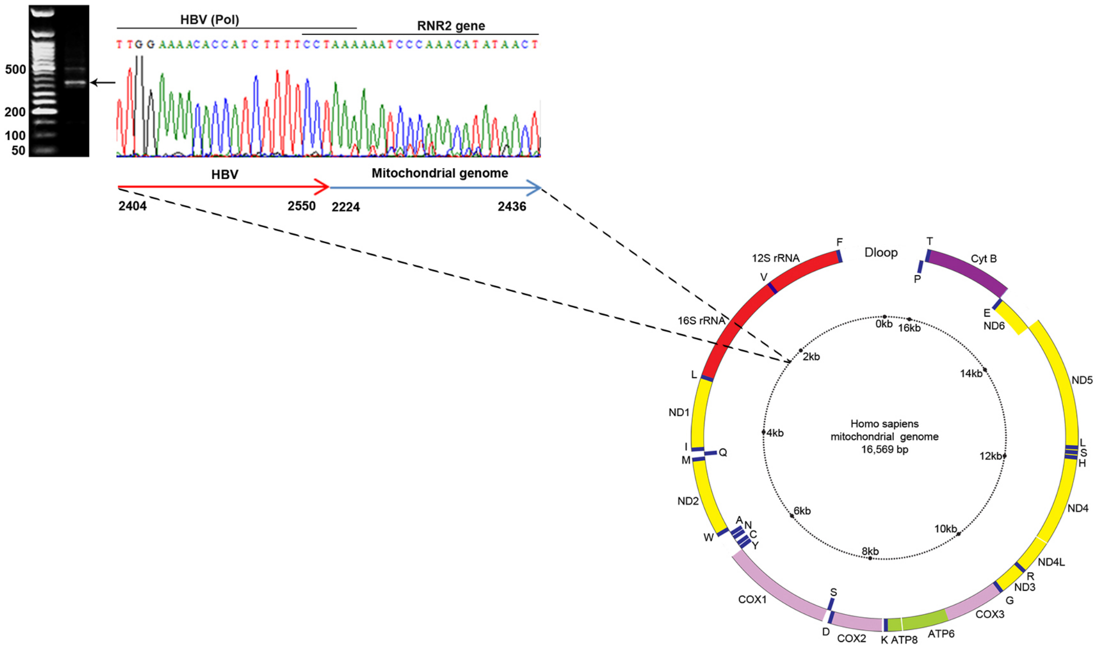


```
  1 TCTCAATCGC CGCGTCGCAG AAGATCTCAA TCTCGGGAAT CTCAATGTTA GTATTCCTTG GACTCATAAG GTGGGGAACT  80
 81 TTACTGGGCT TTATTCTTCT ACTGTACCTG TCTTTAATCC TCATTGGAAA ACACCATCTT TTCCTAAAAA ATCCCAAACA 160
161 TATAACTGAA CTCCTCACAC CCAATTGGAC CAATCTATCA CCCTATAGAA GAACTAATGT TAGTATAAGT AACATGAAAA 240
241 CATTCTCCTC CGCATAAGCC TGCGTCAGAT TAAAACACTG AACTGACAAT TAACAGCCCA ATATCTACAA TCAACCAACA 320
321 AGTCATTATT ACCCTCACTG TCAACCCAAC ACAGG 355
```

b

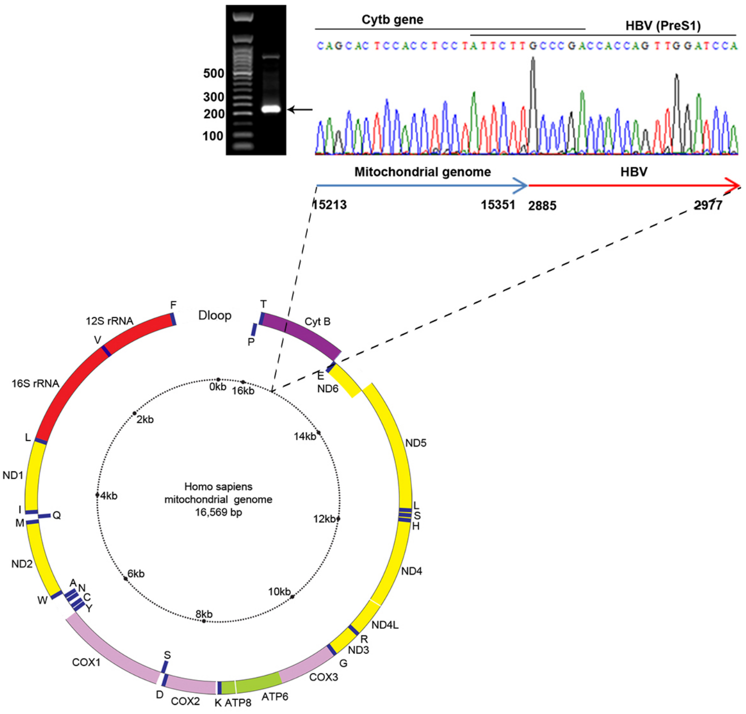


```
  1 TTGGGACAGA CCTAGTTCAA TGAATTTGAG GAGGcTACTC AGTAGACAGT CCCACCCTCA CACGATTCTT TACCTTTCAC  80
 81 TTCATCTTGC CCTTCATTAT TGCAGCCCTA GCAGCACTCC ACCTCCTATT CTTGCCCGAC CACCAGTTGG ATCCAGCCTT 160
161 CAGAGCAAAC ACCGCAAATC CAGATTGGGA CTTCAATACC AACAAGGACC CTTGGCCAGA 220
```

Supplementary Fig. 4. Verification of HBV integration in RNR2 and CYTB genes of mitochondrial DNA from patient 0504 by PCR amplification and Sanger sequencing. Electropherograms detail sequences of the HBV-RNR2 **a** and the HBV-Cytb **b** junctions. The coordinates of the inserted HBV nucleotide fragments are given relative to the reference sequence NC_003977.2. Nucleotide positions at the mitochondrial (GenBank: J01415) insertion sites are also indicated. In the HBV-mitochondrial fusion sequences, HBV nucleotide sequence is shown in red and the mitochondrial sequence in blue. Overlapping microhomology sequence is highlighted in black.

Supplementary Figure 5

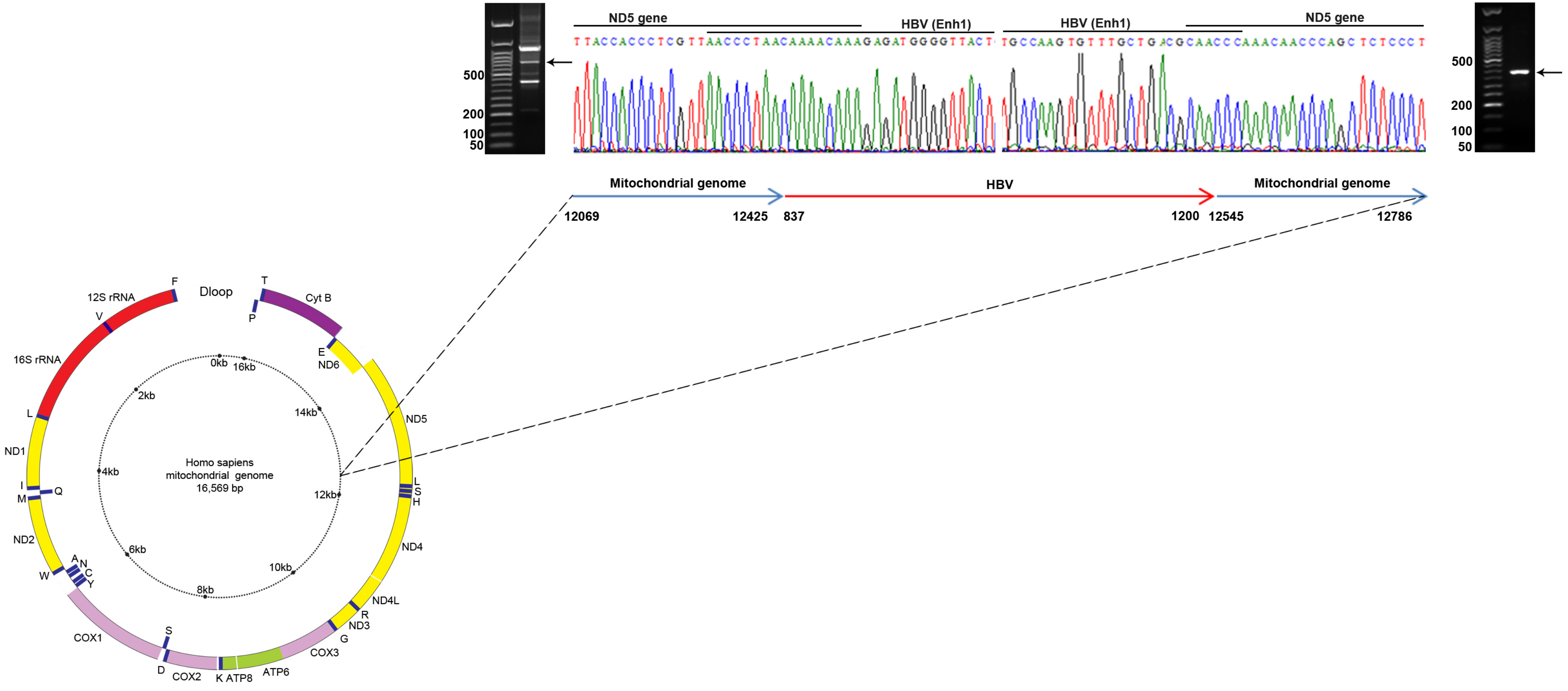

1 TGTTCATACA CCTATCCCCC ATTCTCCTCC TATCCCTCAA CCCCGACATC ATTACCGGGT TTTCCTCTTG TAAATATAGT TTAACCAAAA CATCAGATTG TGAATCTGAC AACAGAGGCT 120

121 TACGACCCCT TATTTACCGA GAAAGCTCAC AAGAACTGCT AACTCATGCC CCCATGTCTA ACAACATGGC TTTCTCAACT TTTAAAGGAT AACAGCTATC CATTGGTCTT AGGCCCCAAA 240

141 AATTTTGGTG CAACTCCAAA TAAAAGTAAT AACCATGCAC ACTACTATAA CCACCCTAAC CCTGACTTCC CTAATTCCCC CCATCCTTAC CACCCTCGTT **AACCCTAACA AAACAAA**GAG 360

361 ATGGGGTTAC TCTCTAAATT TTATGGGTTA TGTCATTGGA TGTTATGGGT CCTTGCCACA AGAACACATC GTACAAAAAA TCAAAGAATG TTTTAGAAAA CTTCCTGTTA ACAGGCCTAT 480

481 TGATTGGAAA GTATTTCAAC GAATTGTGGG TCTTTTGGGT TTTGCTGCCC CTTTCACACA ATGTGGTTAT CCTGCTTTAA TGCCATTGTA TGCATGTATT CAATCGAAGC AGGCTTTCAC 600

601 TTTCTCGCCA ACTTACAAGG CCTTTCTGTG TACACAATAC CTGAACCTTT ACCCCGTTGC CCGGCAACGG CCAGGTCTGT GCCAAGTGTT TGCTGACG**CA ACCC**AAACAA CCCAGCTCTC 720

721 CCTAAGCTTC AAACTAGACT ACTTCTCCAT AATATTCATC CCTGTAGCAT TGTTCGTTAC ATGGTCCATC ATAGAATTCT CACTGTGATA TATAAACTCA GACCCAAACA TTAATCAGTT 840

841 CTTCAAATAT CTACTCATCT TCCTAATTAC CATACTAATC TTAGTTACCG CTAACAACCT ATTCCAACTG TTCATCGGCT GAGAGGGCGT AGGAATTATA 940

Supplementary Fig.5. Verification of HBV integration in *ND5* gene of mitochondrial DNA from patient 0504 by PCR amplification and Sanger sequencing. Electropherogram details the sequence of HBV integration sites in ND5 mitochondrial gene. The coordinates of the inserted HBV nucleotide fragment are given relative to the reference sequence NC_003977.2. Nucleotide positions at the mitochondrial (GenBank: J01415) insertion sites are also indicated. In the HBV-mitochondrial fusion sequence, HBV nucleotide sequence (363bp) is shown in red and mitochondrial sequence in blue. Overlapping microhomology sequence is highlighted in black.

Supplementary Figure 6

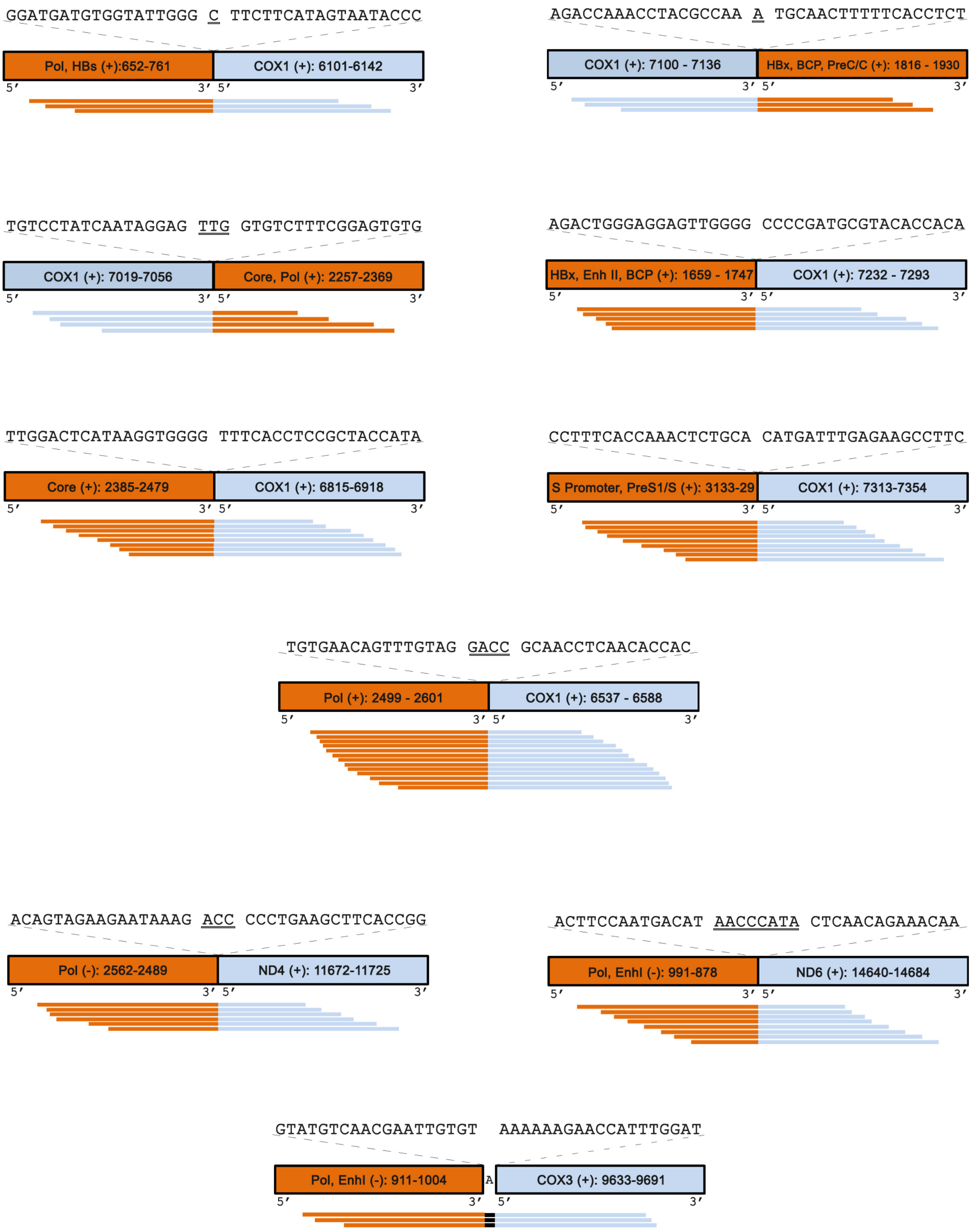

Supplementary Fig.6. Description of viral-mitochondrial chimaeric transcripts detected in HBV-induced HepAD38 cells. The figure highlights unique clipped-sequence fragments of flanking viral-mitochondrial regions with the microhomology underlined.

Supplementary Figure 7

a

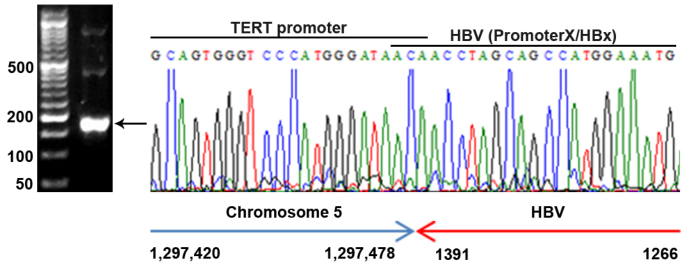


```
  1 CTGGGACCTG AAGCCTGCAG CCTCCCAGCT GCCCCTGCAG TGGGTCCCAT GGGATAACAA CCTAGCAGCC ATGGAAATGA  80
 81 TGTATATTTC CGAGAAAGGA CGACAGAATT ATCAGTCCCA ATGAGTTTTG CTCCAGACCG GCTGCGAGCA AAACAAGCTG 160
161 CAAGGAGTTC CGCAGTATGG AT 182
```

b

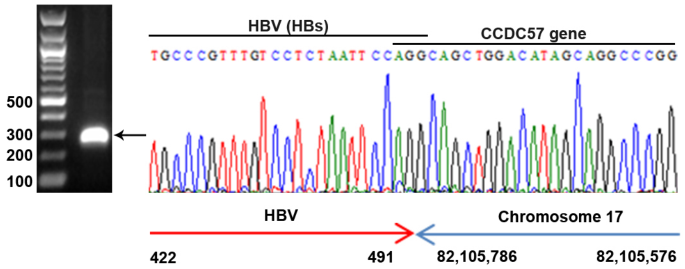


```
  1 TATGCCTCAT CTTCTTGTTG GTTCTTCTGG ACTATCAAGG TATGTTGCCC GTTTGTCCTC TAATTCCAGG CAGCTGGACA  80
 81 TAGCAGGCCC GGAGTGTCCC CCTGTGGAGG GACCGTGGGA ACCAGATCAC GTGGGCCTCC CTCCCCCTGT ACCCCATTGC 160
161 TCTCCTGTTG TCACACTCAG TCCCACAAAG GCAGCCCCGG GGCCACGGTG ACCCCCCAGG GCTGAGCTCC AGGAGCTCAG 240
241 GGGTGGGGGT GTCCATTAGG AGACTTTAGC AGGTGAGGA 279
```

Supplementary Fig.7. Verification of HBV integration in the promoter of the TERT gene and in the CCDC57 gene of PLC/PRF/5 cells by PCR amplification and Sanger sequencing. Electropherograms details the sequence of the HBV (red) integration site **a** in the TERT promoter (blue) and **b** in the CCDC57 gene (blue). Overlapping microhomology sequence is highlighted in black.

Supplementay Figure 8

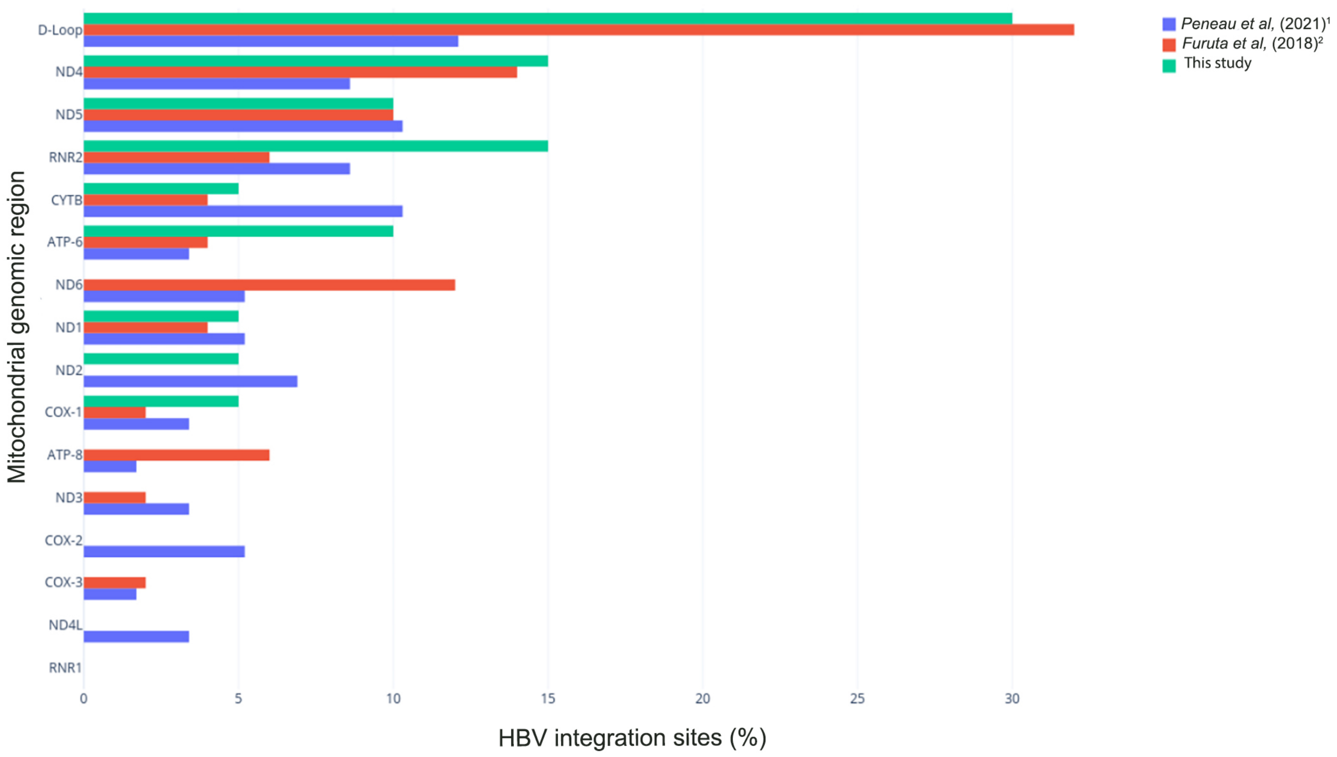

Supplementary Fig. 8. Distribution of HBV integration sites in the genes and in the *D-loop* regulatory region of mitochondria isolated from liver tissue specimens, by three different studies (blue bar: Peneau et al[1]; Red bar: Furuta et al[2]; Green bar: Giosa et al, this study)

Supplementary Figure 9. Uncropped gel images related to Figure 4a, 4b, and 4c.

Figure 4
a) Full-length
HBV DNA
(Mitochondrial
etracts)

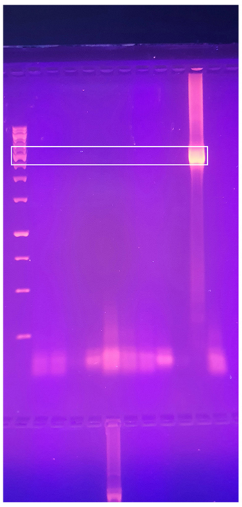

Figure 4
a) HBV cccDNA
(Mitochondrial
extracts)

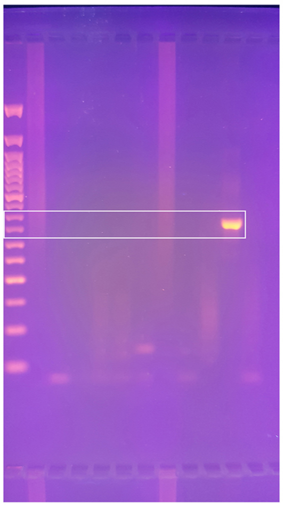

Figure 4
b) Full-length
HBV DNA
(Mitochondrial
extracts)

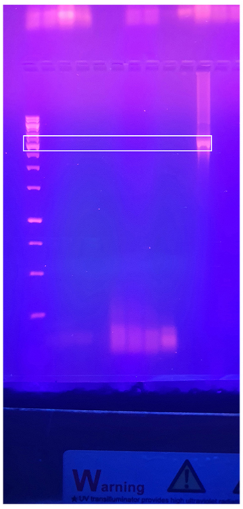

Figure 4
b) HBV cccDNA
(Mitochondrial
extracts)

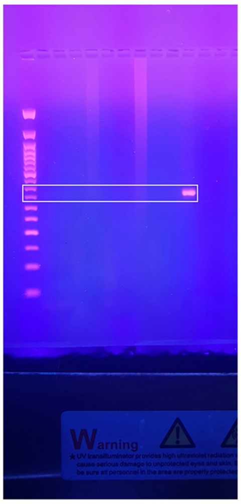

Figure 4
c) Full-length
HBV DNA
(Nuclear extracts)

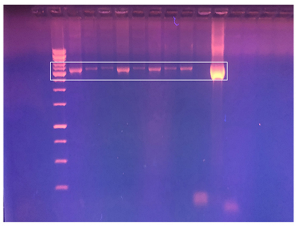

Figure 4
c) HBV cccDNA
(Nuclear extracts)

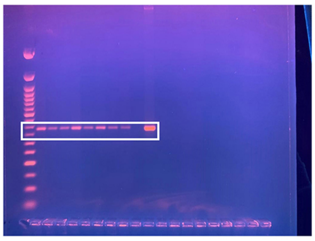

Figure 4
d) Full-length
HBV DNA
(Nuclear extracts)

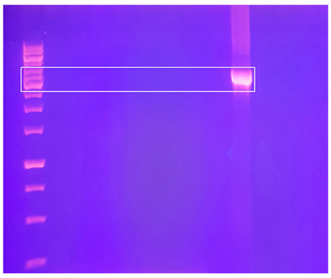

Figure 4
d) HBV cccDNA
(Nuclear extracts)

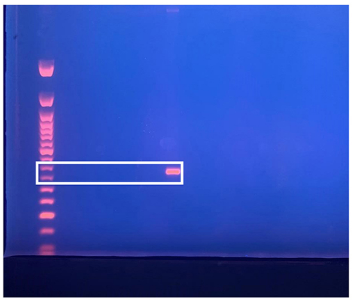

Supplementary Figure 10. Uncropped gel images related to Figure 4e.

Figure 4
e) HBV preS1

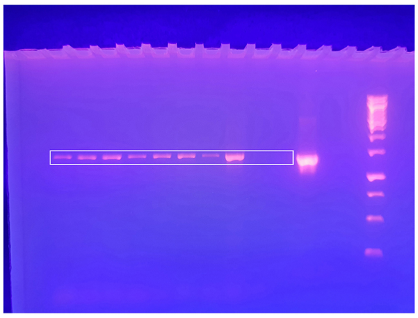

Figure 4
e) HBV S

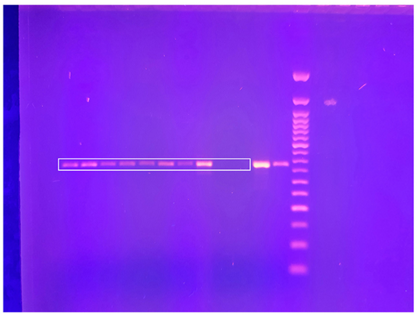

Figure 4
e) HBV X

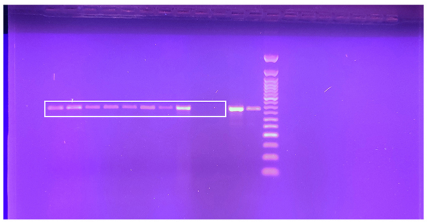

Figure 4
e) HBV Epsilon

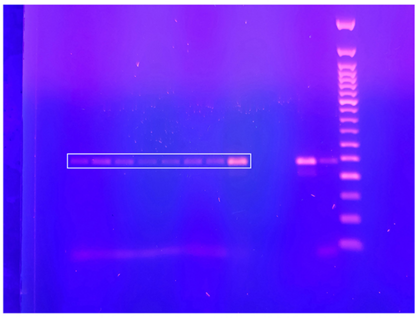

Figure 4
e) COX3

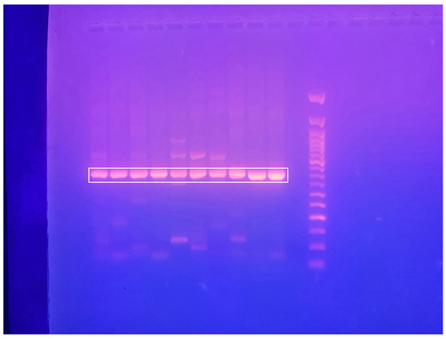

Figure 4
e) GAPDH

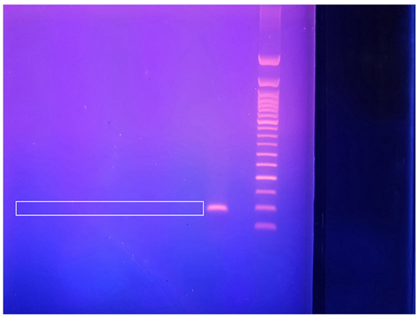

Supplementary Figure 11. Uncropped WB and X-ray film images related to Figure 5b and 5c.

Figure 5
b) PNPase

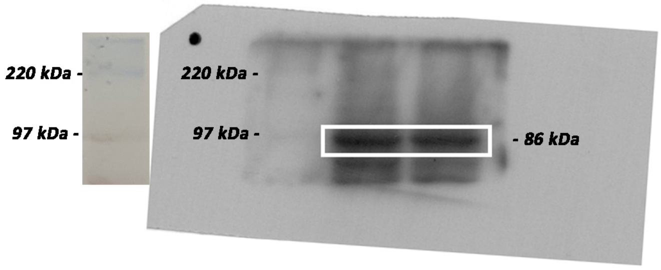

Figure 5
b) β-tubulin

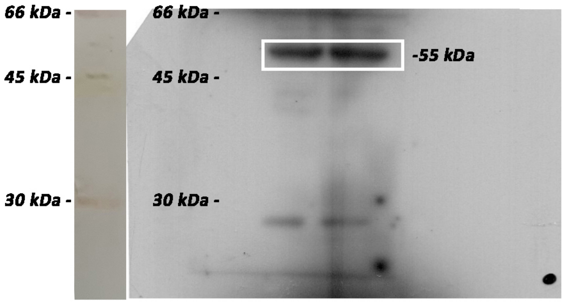

Figure 5
c) Import

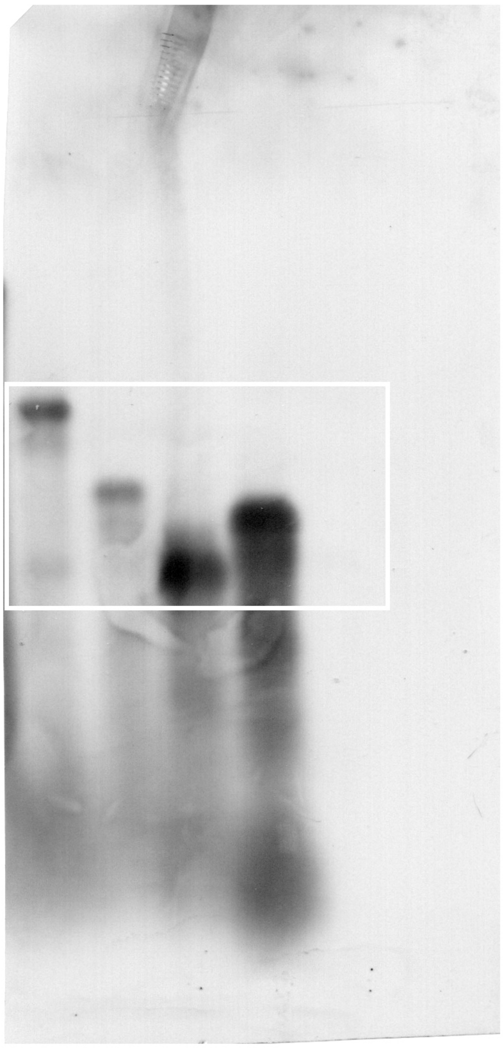

Figure 5
c) HBV RN/
Input

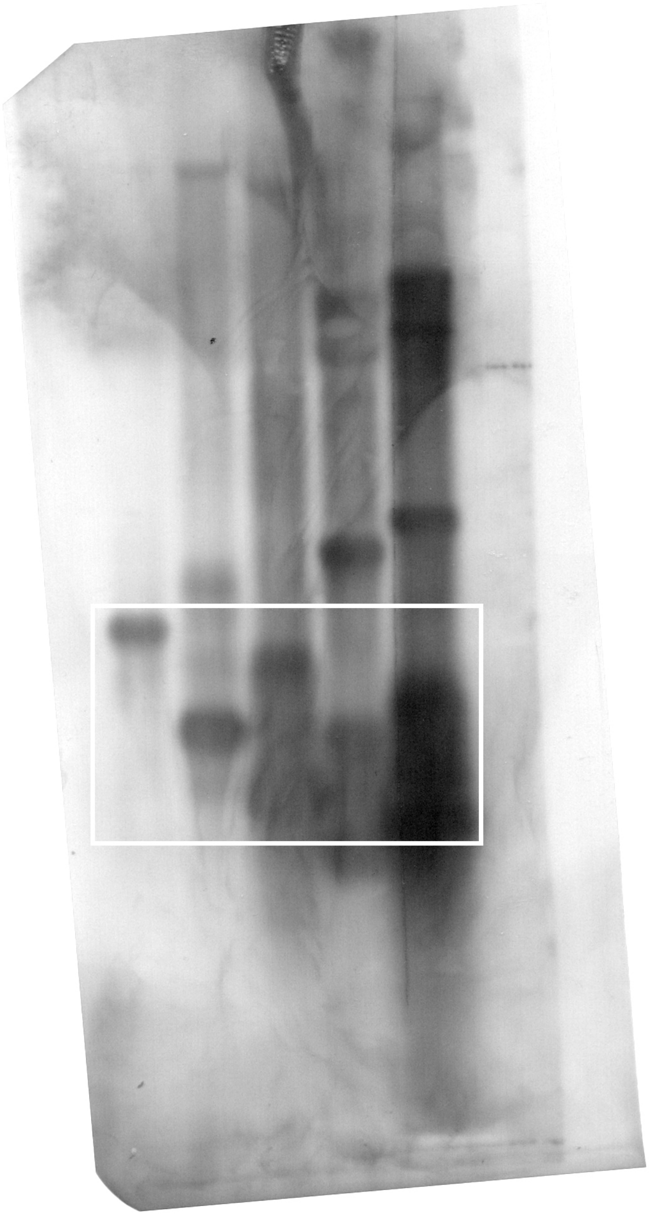

Supplementary Figure 12. Uncropped WB images related to Figure 6a.

Figure 6
a) PNPase

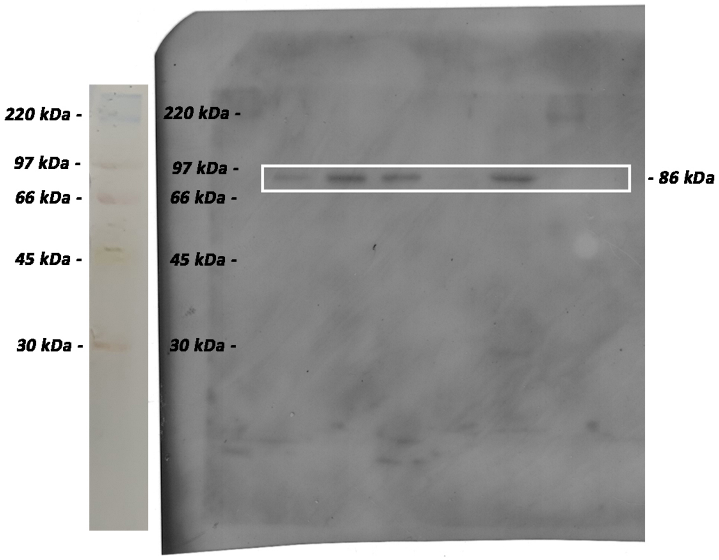

Figure 6
a) Mortalin

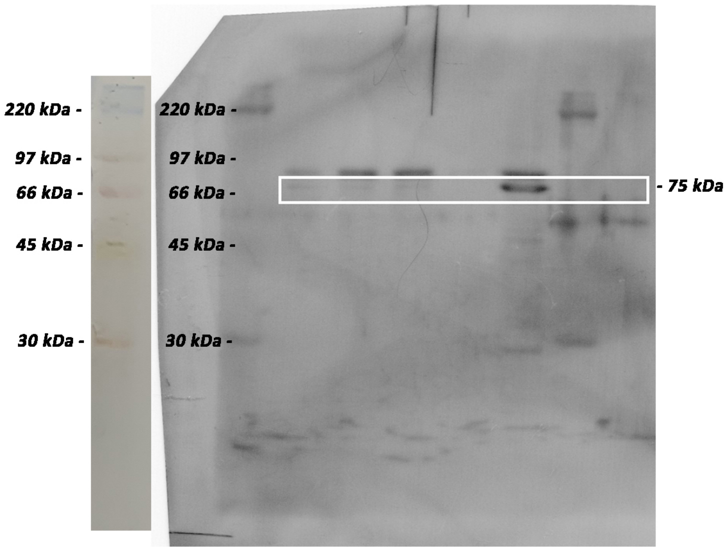

Figure 6
a) HSP90

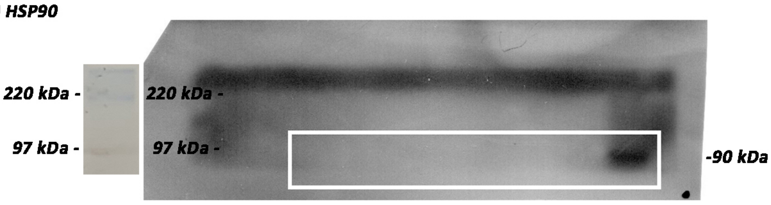

Figure 6
a) β-tubulin

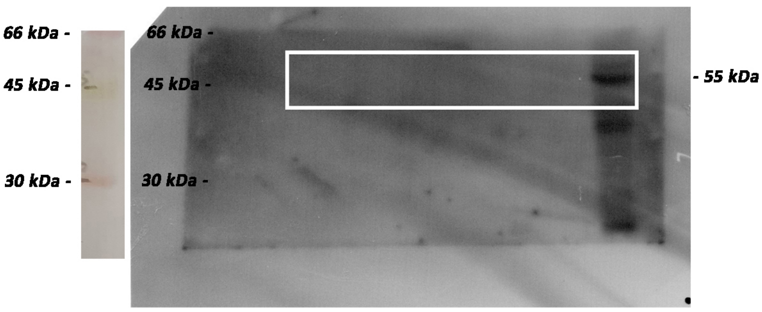

Supplementary Figure 13. Uncropped gel images related to Supplementary Figure 3a, 3b, 4a, 4b, 5, 7a, and 7b.

Supplementary Figure 3a

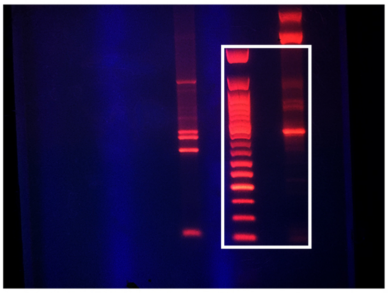

Supplementary Figure 3b

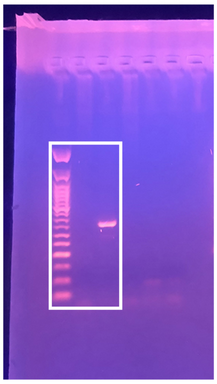

Supplementary Figure 4a

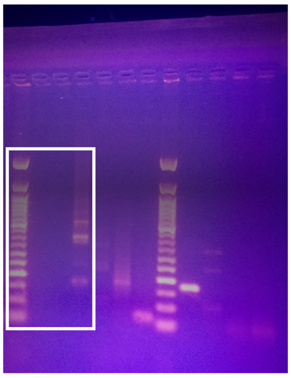

Supplementary Figure 4b

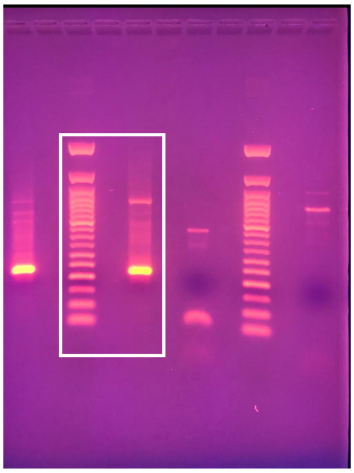

Supplementary Figure 5

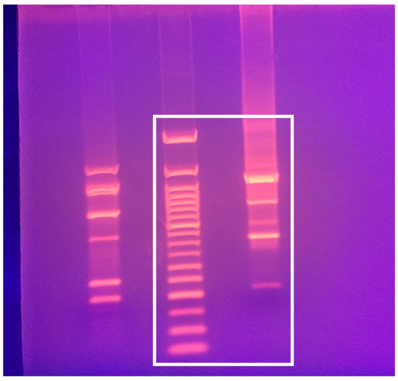

Supplementary Figure 5

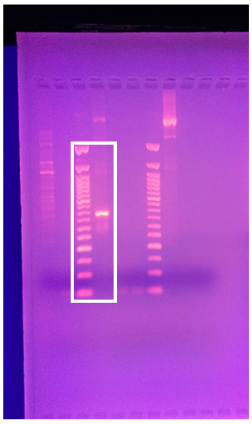

Supplementary Figure 7a

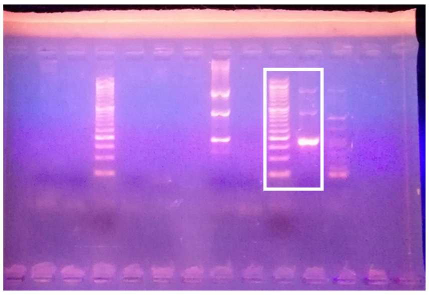

Supplementary Figure 7b

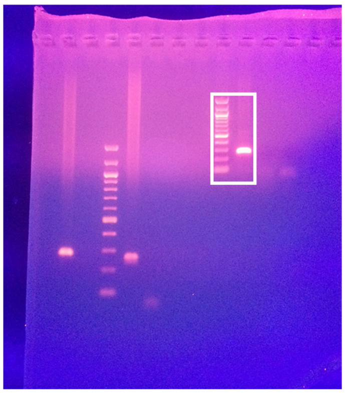

Supplementary References

1. Peneau, C. et al. Hepatitis B virus integrations promote local and distant oncogenic driver alterations in hepatocellular carcinoma. *Gut* (2021).
2. Furuta, M. et al. Characterization of HBV integration patterns and timing in liver cancer and HBV-infected livers. *Oncotarget* **9**, 25075-25088 (2018).
