# Supplementary material for: Mitochondrial DNA is a target of HBV integration
Source: Commun Biol. 2023 Jul 3;6:684. doi: 10.1038/s42003-023-05017-4 (PMC10318008; doi:10.1038/s42003-023-05017-4)
Supplement: Supplementary file 3 — Description of Additional Supplementary Files [file 42003_2023_5017_MOESM3_ESM.pdf]

# Description of Additional Supplementary Files

**File name:** Supplementary Data 1

**Description:** The source data behind the graph shown in Supplementary Figure 1 and the Circos plot shown in Supplementary Figure 2 of the paper

**File name:** Supplementary Data 2

**Description:** The source data behind the results reported in the text of the manuscript

**File name:** Supplementary Data 3

**Description:** The source data behind the Circos plot shown in Figure 3 of the paper

**File name:** Supplementary Data 4

**Description:** The source data behind the results represented in Supplementary Figure 6 and the results reported in the text of the manuscript.

**File name:** Supplementary Data 5

**Description:** The source data behind the graph shown in Supplementary Figure 8 the results reported in the text of the manuscript
