# Supplementary material for: Mitochondrial DNA is a target of HBV integration
Source: Commun Biol. 2023 Jul 3;6:684. doi: 10.1038/s42003-023-05017-4 (PMC10318008; doi:10.1038/s42003-023-05017-4)
Supplement: Supplementary file 9 — Reporting Summary [file 42003_2023_5017_MOESM9_ESM.pdf]

nature portfolio

Corresponding author(s): Teresa Pollicino

Last updated by author(s): May 20, 2023

Reporting Summary

Nature Portfolio wishes to improve the reproducibility of the work that we publish. This form provides structure for consistency and transparency in reporting. For further information on Nature Portfolio policies, see our Editorial Policies and the Editorial Policy Checklist.

Statistics

For all statistical analyses, confirm that the following items are present in the figure legend, table legend, main text, or Methods section.

n/a	Confirmed	
☐	☒	The exact sample size (*n*) for each experimental group/condition, given as a discrete number and unit of measurement
☐	☒	A statement on whether measurements were taken from distinct samples or whether the same sample was measured repeatedly
☐	☒	The statistical test(s) used AND whether they are one- or two-sided<br>*Only common tests should be described solely by name; describe more complex techniques in the Methods section.*
☒	☐	A description of all covariates tested
☒	☐	A description of any assumptions or corrections, such as tests of normality and adjustment for multiple comparisons
☐	☒	A full description of the statistical parameters including central tendency (e.g. means) or other basic estimates (e.g. regression coefficient) AND variation (e.g. standard deviation) or associated estimates of uncertainty (e.g. confidence intervals)
☐	☒	For null hypothesis testing, the test statistic (e.g. *F*, *t*, *r*) with confidence intervals, effect sizes, degrees of freedom and *P* value noted<br>*Give P values as exact values whenever suitable.*
☐	☒	For Bayesian analysis, information on the choice of priors and Markov chain Monte Carlo settings
☒	☐	For hierarchical and complex designs, identification of the appropriate level for tests and full reporting of outcomes
☒	☐	Estimates of effect sizes (e.g. Cohen's *d*, Pearson's *r*), indicating how they were calculated

Our web collection on statistics for biologists contains articles on many of the points above.

Software and code

Policy information about availability of computer code

Data collection	The sequencing data reported in this paper have been deposited in the NCBI SRA database (DNAseq accession [PRJNA650273]; SRA Study: SRP283983)
Data analysis	Illumina raw paired-end reads (2x250 bp) were processed to remove adapters and low-quality bases with the Trimmomatic program (v.0.36). BWA software (version: 0.7.12-r1039) was used to align the clean reads. The Picard tool (version 2.22.0) (http://broadinstitute.github.io/picard) was employed to remove optical duplicate reads from the BAM files; SAMtools software was utilised to extract chimaeric reads (Li, Handsaker et al. 2009). BEDTools utility (Quinlan 2014) was used to extract primary and secondary mapping coordinates to properly count the number of integration events occurring in the hybrid genome (the flag -cigar was used in the bamtobed tool)The resulting genomic coordinates, including chromosomal locations related to the integration of HBV-DNA into the human genome, were then used to retrieve reads related to each integration event in order to reconstruct the chimeric sequences using Cap3 (Huang and Madan 1999) and cd-hit (Fu, Niu et al. 2012) software. Assembled chimeras were mapped back to the hybrid genome using the BLAST algorithm (Camacho, Coulouris et al. 2009) with the following options: task=blastn-short, dust=no, soft_masking=false, word_size=7, penalty=-3, reward=2, gapopen=5, gapextend=2. Microhomology was determined by identifying the DNA mapping in each chimera on both human and HBV genomes respectively. All the bioinformatics tools described above have been integrated into an automatic computational pipeline for accurate and efficient detection of viral integration events in the human genome that is available at the GitHub repository (https://github.com/DomeJoyce/HBVIF)

For manuscripts utilizing custom algorithms or software that are central to the research but not yet described in published literature, software must be made available to editors and reviewers. We strongly encourage code deposition in a community repository (e.g. GitHub). See the Nature Portfolio guidelines for submitting code & software for further information.

Data

Policy information about availability of data

All manuscripts must include a data availability statement. This statement should provide the following information, where applicable:

- Accession codes, unique identifiers, or web links for publicly available datasets
- A description of any restrictions on data availability
- For clinical datasets or third party data, please ensure that the statement adheres to our policy

Accession code, Web Link: https://github.com/DomeJoyce/HBVIF (GitHub repository)
Datasets of this study are freely available. The sequencing data reported in this paper have been deposited in the NCBI SRA database (DNAseq Accession: PRJNA650273; SRA Study: SRP283983). The authors declare that all other data supporting the findings of this study are available within the article and its Supplementary Information files or are available from the corresponding author on reasonable request.

Human research participants

Policy information about studies involving human research participants and Sex and Gender in Research.

Reporting on sex and gender	Sex was not considered in the study design since all the previously published studies have reported that HBV integration into the host genome occurs equally in men and women
Population characteristics	See above
Recruitment	We included in the study all the tumour and non-tumour liver tissue specimens from HBsAg-positive patients with HCC that were stored at -80°C at the Laboratory of Molecular Hepatology of the University of Messina, Italy, and that were available to be analysed.
Ethics oversight	The collection and processing of all samples was approved by the Ethics Committee of the Messina University Hospital (protocol number 64/15), Italy.

Note that full information on the approval of the study protocol must also be provided in the manuscript.

Field-specific reporting

Please select the one below that is the best fit for your research. If you are not sure, read the appropriate sections before making your selection.

☒ Life sciences ☐ Behavioural & social sciences ☐ Ecological, evolutionary & environmental sciences

For a reference copy of the document with all sections, see nature.com/documents/nr-reporting-summary-flat.pdf

Life sciences study design

All studies must disclose on these points even when the disclosure is negative.

Sample size	The study had as main aim the development of a high-throughput HBV integration sequencing (HBIS) method that could allow for sensitive identification of HBV integration sites and enumeration of integration clones. Therefore, our research was preliminary and exploratory rather than definitive and final. For this reason, no sample-size calculation was performed.Tumour tissues from 7 patients and paired adjacent non-tumour tissues from 6 of them were studied. Moreover, frozen liver biopsy specimens from 5 patients with HBeAg-negative chronic hepatitis B (CHB) and 8 frozen liver biopsy specimens from HBsAg-negative patients with chronic liver disease (CLD) were included in this study. For in vitro experimens conducted on cell lines, sample size was determined based on general standards for biological studies and requirements for statistical analysis, attempting to have a minimum of n=3 biological replicates with sufficient reproducibility.
Data exclusions	No data were excluded from the analyses.
Replication	All attempts at replication were successful. Some findings were not replicated because we had a very limited amount of human liver tissue for each patient.
Randomization	Allocation into experimental groups was not relevant to our study.
Blinding	Blinding was not relevant to our study as samples/libraries were anonymised and processed in a randomised order

Reporting for specific materials, systems and methods

We require information from authors about some types of materials, experimental systems and methods used in many studies. Here, indicate whether each material, system or method listed is relevant to your study. If you are not sure if a list item applies to your research, read the appropriate section before selecting a response.

Materials & experimental systems

n/a	Involved in the study
☐	☒ Antibodies
☐	☒ Eukaryotic cell lines
☒	☐ Palaeontology and archaeology
☒	☐ Animals and other organisms
☒	☐ Clinical data
☒	☐ Dual use research of concern

Methods

n/a	Involved in the study
☒	☐ ChIP-seq
☒	☐ Flow cytometry
☒	☐ MRI-based neuroimaging

Antibodies

Antibodies used	Rabbit anti-PNPase (catalog number GTX118737, GeneTex); rabbit anti-Mortalin (cat. n. 3593, Cell Signaling Technology); anti-β Tubulin mouse (cat. n. 86298, Cell Signaling Technology), mouse anti-Hsp90 (cat. n. ab13492, Abcam)
Validation	Rabbit anti-PNPase catalog number GTX118737, GeneTex Website Reference: Wang DD et al. J Biol Chem 2014; Helicase SUV3, Polynucleotide Phosphorylase PNPase, and Mitochondrial Polyadenylation Polymerase mtPAP Form a Transient Complex to Modulate Mitochondrial mRNA Polyadenylated Tail Lengths in Response to Energetic Changes; Rabbit anti-Mortalin cat. n. 3593, Cell Signaling Technology), Website Reference: Li M. et al Blood Cancer Discov 2022;3(1):50-65. Translational Activation of ATF4 through Mitochondrial Anaplerotic Metabolic Pathways Is Required for DLBCL Growth and Survival; anti-β Tubulin mouse (cat. n. 86298, Cell Signaling Technology Website Reference: Andrea Lopez, et. al Nature Commun 2022; 7,13(1):1199. Co-targeting of BAX and BCL-XL proteins broadly overcomes resistance to apoptosis in cancer. Mouse anti-Hsp90, cat. n. ab13492, Abcam Website Reference: Dekel E et al. 20S proteasomes secreted by the malaria parasite promote its growth. Nat Commun 12:1172 (2021).

Eukaryotic cell lines

Policy information about cell lines and Sex and Gender in Research

Cell line source(s)	The PLC/PRF/5 human hepatoma cell line (SIGMA, catalogue number 85061113, Lot Number:10D004); the HepG2 cell line (ATCC, catalogue number HB-8065TM); the Vero cell line (provided by Prof Maria Teresa Sciortino); The HepAD38 cell line (kindly provided by Dr. Cristopher Seeger)
Authentication	None of the cell lines used was authenticated
Mycoplasma contamination	I confirm that all cell lines tested negative for mycoplasma contamination.
Commonly misidentified lines (See ICLAC register)	No misidentified cell line was used
